# Low-Profile Suture Button Technique with Additional AC Cerclage for High-Grade Acromioclavicular Joint Dislocations: A Retrospective Outcome Analysis

**DOI:** 10.3390/jcm14196888

**Published:** 2025-09-29

**Authors:** Larissa Eckl, Philipp Vetter, Frederik Bellmann, Jonas Pawelke, Doruk Akgün, Philipp Moroder, Asimina Lazaridou, Markus Scheibel

**Affiliations:** 1 Sektion Sportorthopädie, Technical University (TU)-Klinikum, 81675 Munich, Germany; larissa.eckl@mri.tum.de (L.E.); philipp.moroder@kws.ch (P.M.); 2 Shoulder and Elbow Surgery, Schulthess Clinic, 8008 Zurich, Switzerland; p.vetter.med@gmail.com (P.V.); frederik.bellmann@kws.ch (F.B.); 3 Unfall-, Hand- und Wiederherstellungschirurgie, Universitätsklinikum Gießen und Marburg, 35392 Giessen, Germany; jonas.pawelke@med.uni-giessen.de; 4 Centrum für Muskuloskeletale Chirurgie, Charité Universitätsmedizin Berlin, 13353 Berlin, Germany; doruk.akguen@charite.de; 5 Research Group Upper Extremities & Hand, Schulthess Clinic, 8008 Zurich, Switzerland; asimina.lazaridou@kws.ch

**Keywords:** acromioclavicular joint, arthroscopic surgical procedures, suture anchors, joint instability

## Abstract

**Background:** For high-grade dislocation of the acromioclavicular (AC) joint, surgical treatment is widely recommended. This study aimed to evaluate the clinical and radiological outcomes after arthroscopic-assisted stabilization of acute high-grade AC joint dislocations using a low-profile suture button (LPSB) combined with percutaneous AC cerclage fixation. A secondary objective was to quantify clavicular tunnel widening (cTW) and explore its correlation with clinical and radiological outcomes. **Methods:** This retrospective study included 45 patients with acute Rockwood type V injuries treated with the LPSB technique and additional AC cerclage. Clinical outcomes were the Constant Score (CS), Subjective Shoulder Value (SSV), Taft Score (TF), AC Joint Instability Score (ACJI), and VAS for pain upon palpation. Radiological assessment included coracoclavicular (CC) distance and percentage deviations compared to the contralateral side, reclassified according to Rockwood, dynamic posterior translation (DPT), cTW measurements, and assessment of ossifications and AC joint osteoarthritis. **Results:** After 35.3 months, significant improvements were observed in CC distance and percentage deviation. A total of 27.3% were reclassified as Rockwood type III and 2.3% as type V. Initial overreduction persisted in 18.2%. DPT was observed in 34.1% of cases. The mean CS was 89.64, the SSV was 91.1, and the VAS was 0.8. cTW occurred only below the superior button and increased significantly over time, showing a negative correlation with the SSV but no correlation with any radiological outcome parameter. No implant-related revision surgery was reported. **Conclusions:** Arthroscopic-assisted stabilization of acute high-grade AC joint dislocations using the LPSB technique with AC cerclage fixation provides excellent clinical outcomes and high patient satisfaction, with minimal implant-related complications and no need for revision surgery due to implant issues. Although cTW occurs, its clinical impact appears limited within this procedure.

## 1. Introduction

**Figure 1:**
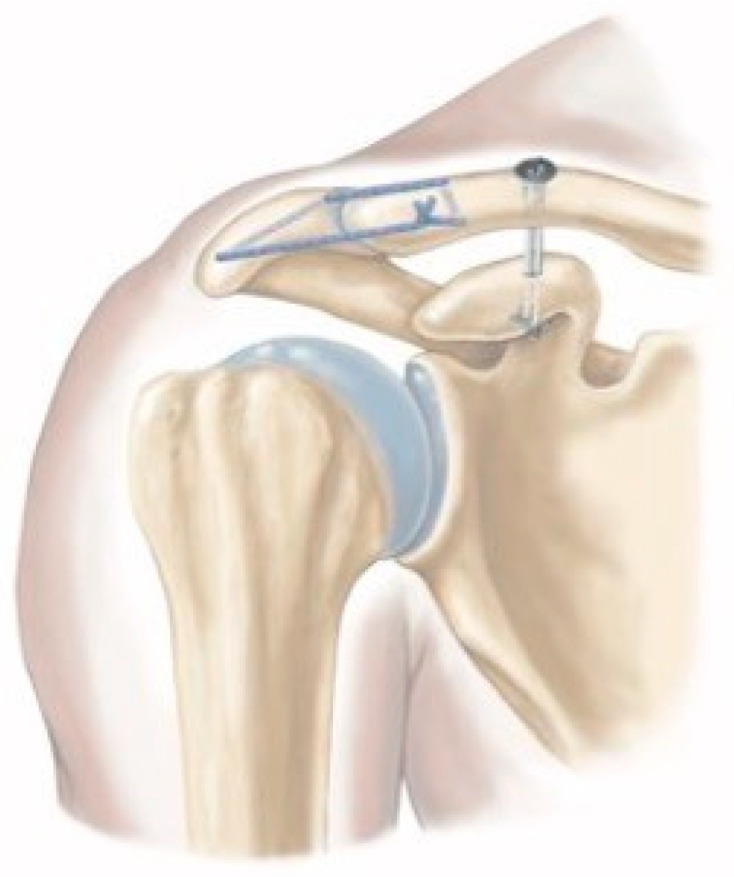
Schematic illustration of the low-profile TightRope™ (LPSB) system, designed to reduce clavicular tunnel widening and prevent knot stack above the superior implant, combined with an additional AC cerclage fixation [25].

Dislocation of the acromioclavicular (AC) joint is one of the most common shoulder injuries in adults and frequently affects young, athletically active people [1,2]. Evaluation of the extent of injury is based on radiographic findings, and it is commonly classified according to the Rockwood classification system [3]. Conservative treatment is recommended for low-grade dislocations, while surgical stabilization is widely used for high-grade dislocations [4,5,6,7,8]. There is ongoing debate about the necessity of operative treatment for Rockwood type III and V injuries, as conservative management has shown comparable outcomes [9,10,11,12]. In the event of surgery, arthroscopic and arthroscopic-assisted techniques offer numerous advantages over open procedures, such as diagnosis and treatment of concomitant glenohumeral pathologies, no need for secondary removal of the hardware, significantly better rates of return to sport, better cosmetic results and an overall higher subjective patient satisfaction [13,14,15]. Within the various amount of arthroscopic and arthroscopic-assisted treatment options, pulley-like suture implants are recommended for vertical stabilization of the AC joint [8,16,17,18]. Moreover, biomechanical research has demonstrated the benefit of additional stabilization in the horizontal plane to achieve sufficient stability equivalent to the native AC joint [19,20,21]. Despite generally good clinical outcomes, these minimally invasive techniques also exhibit complications such as implant-associated discomfort due to knot stacking, vertical and horizontal loss of reduction, clavicular tunnel widening (cTW) caused by micromovements at direct suture–bone contact surfaces and drill-hole-associated fractures [14,15,16,18,22,23,24]. The special design of the low-profile suture button (LPSB) aims to minimize knot stack by lowering the sutures and to reduce cTW by guiding the sutures within the first few millimeters of clavicular drilling (Figure 1). The primary purpose of this study was to evaluate the clinical and radiological outcomes after arthroscopic-assisted stabilization of acute high-grade AC joint dislocation using an LPSB with additional percutaneous AC cerclage fixation. The secondary objective was to quantify cTW and explore its correlation with clinical and radiological outcomes.

## 2. Materials and Methods

### 2.1. Ethics Approval

This study was approved by the local ethics committee (BASEC 2021-00675) and institutional review board (EA1/298/12) in conjunction with the 1964 Helsinki Declaration and its later amendments or comparable ethical standards. This study is a retrospective outcome analysis of previously collected patient data and is therefore classified as level IV evidence.

### 2.2. Patient Selection

Patients with acute (less than three weeks after reported trauma) AC joint dislocation classified as Rockwood type V and treated using a LPSB stabilization procedure with additional percutaneous AC cerclage fixation were included in this retrospective analysis. All patients were selected from two local shoulder surgery clinic databases and treated between 2018 and 2021. Exclusion criteria was previous surgery to the same shoulder, concomitant fractures, and follow-up (FU) less than 24 months after surgery, as well as patients with injuries other than Rockwood type V. All patients were invited for a final FU assessment, which included a radiographic examination, a physical examination of both shoulders and a questionnaire for clinical outcome analysis. These assessments were performed by an independent observer. The patients were treated according to this standardized procedure by several surgeons (M.S., P.M., D.A.).

### 2.3. Radiological Evaluation

**Figure 2:**
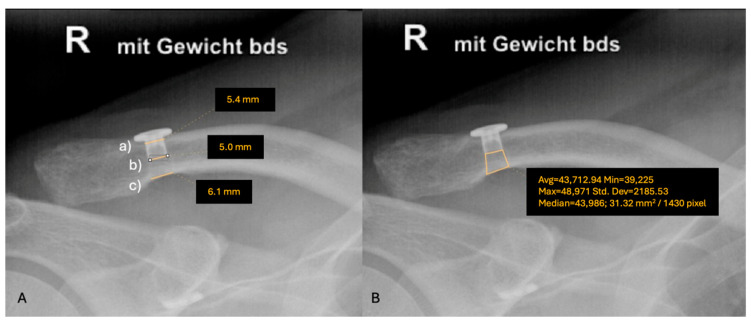
Assessment of clavicular tunnel widening (cTW), with (**A**) illustrating the measurement of the tunnel diameter at the superior cortex (a), the inferior border of the metal inlet (b), and the inferior cortex (c) and (**B**) demonstrating the measurement of the sub-button area (mm^2^) using the polygon tool.

On panoramic anteroposterior (a.p.) stress radiographs with a 10 kg load on each side, the CC distance was measured on both the affected and contralateral sides preoperatively, and the mean percentage difference compared to the contralateral side was calculated. The CC distance and the mean percentage difference were measured again after 24 months and, based on the percentage difference, reclassified according to the Rockwood classification as follows: type I was classified as having an equivalent CC distance on both sides. A type II injury was classified as having a CC distance increase of less than 25%. A type III injury was characterized by a CC distance increase of 25–100%, and a type V injury was classified as having a CC distance increase of 100% or more [3]. Horizontal loss of reduction was assessed on Alexander views and reported as dynamic posterior translation (DPT), which was classified as none, partial, or complete [26]. For cTW analysis, a postoperative radiographic image was taken for comparison with the follow-up results. To assess the cTW, the tunnel diameter at the superior cortex, the inferior border of the metal inlet and inferior cortex were measured (Figure 2A). To calculate the sub-button area (mm^2^), the polygon tool (JiveX Diagnostic 5.3.0.7) was applied inferior to the metal inlet (Figure 2B) to quantify the 3-dimensional widening process. Ossifications between the clavicle and coracoid process, as well as degenerative changes in the AC joint, were assessed [27].

### 2.4. Clinical Evaluation

Clinical outcome scores included the Constant Score (CS), Subjective Shoulder Value (SSV), Taft Score (TF), and Acromioclavicular Joint Instability Score (ACJI) [26,28,29]. Moreover, the VAS for pain upon palpation directly above the superior implant was assessed.

### 2.5. Surgical Treatment

The image-intensifier-controlled and arthroscopic-assisted bidirectional LPSB technique was performed as described before [30]. Under general anesthesia, the patient was placed in a beach chair position. A standard posterior viewing portal, a lateral viewing portal, an anteroinferior working portal and an incision of 2 to 3 cm at the top of the clavicle were established. Diagnostic arthroscopy via the posterior viewing portal to detect and treat concomitant glenohumeral pathologies was done first. To achieve completely clear visualization of the undersurface of the coracoid before placing the coracoclavicular drill channel, the base of the coracoid and the subcoracoid bursa were dissected. A marking hook was then placed underneath the prepared coracoid arch. Under arthroscopic guidance, 3-mm transclavicular-transcoracoid drilling was performed between the anatomic attachments of the trapezoid and conoid ligament using a drill bit with an implemented Kirschner wire (K-wire). After controlling the correct placement of the drilling by using the image intensifier, the superior clavicle was unicortically overdrilled with a 5.1-mm drill bit. The K-wire was then replaced by a nitinol suture passing wire, which was retrieved through the anteroinferior portal. For triangular percutaneous AC cerclage fixation, acromial and clavicular drill holes were established with the help of a marking hook and under image intensifier control. Transclavicular drilling from the anterior to the posterior was carried out, and a 1.25-mm K-wire was inserted, which was then overdrilled with a cannulated 2.7-mm drill bit and replaced by a nitinol suture passing wire. For transacromial drilling from lateral–caudal to medial–cranial, another 1.25-mm K-wire was used and, after overdrilling with a cannulated 2.7-mm drill bit, also replaced by a nitinol suture passing wire. Within this step, the clavicle was casualized to avoid transarticular drilling. After all drilling was completed, the sutures of a low-profile TightRope^®^ implant (Arthrex, Naples, FL, USA) were attached to the proximal eyelet of the first nitinol suture passing wire and pulled through the clavicle and the coracoid. A Dog Bone^®^ button (Arthrex, Naples, FL, USA) was then attached to the inferior sutures, and the correct position underneath the coracoid arch was reached under arthroscopic guidance by pulling the upper end of the sutures in an alternating manner. By alternately pulling the sutures, the superior button reached its final epicortical position and facilitated the anatomic reduction in the ACJ. With 80–100 N, a suture tensioner was used to ensure sufficient stability. To complete the AC cerclage, nonabsorbable tape was attached to the clavicular nitinol suture passing wire and pulled through the clavicular incision. Next, the tape was attached to the loop of the acromial suture passing wire and retracted via the lateral portal. At the anteroinferior portal, a knot pusher was connected to the tape, and the tape was retrieved via the clavicular incision. The lateral end of the tape was attached to the knot pusher and retrieved via the clavicular incision in the same manner. The ends of the tape were strongly tightened and knotted. The final step included an arthroscopic inspection and image intensifier control to ensure correct placement of the device. The sutures were securely knotted, cut, and lowered into the recessed hole of the LPSB to prevent future knot stack. The deltotrapezoidal fascia was reconstructed, and the clavicular incision and portals were closed in a standard fashion.

### 2.6. Rehabilitation Protocol

Postoperative treatment followed a standardized rehabilitation protocol, beginning with six weeks of immobilization of the injured shoulder in an abduction brace. During the first three weeks, passive range of motion was restricted to flexion and abduction up to 45°. By the sixth week, passive motion was increased to 90°. Starting in the seventh week, free passive and early active range of motion exercises were introduced. Strengthening exercises were allowed from the tenth postoperative week onward.

### 2.7. Statistical Analysis

Statistical analysis was performed using IBM SPSS^®^ Statistics (version 20.0.1.0; IBM Corp., Armonk, NY, USA). The Shapiro–Wilk test was used to assess the normality of data distributions. Differences between preoperative, postoperative, and follow-up measurements were analyzed using the Friedman test for repeated measures, with pairwise comparisons conducted via the Wilcoxon signed-rank test. For normally distributed paired data, a paired-samples *t*-test was used, while the Wilcoxon signed-rank test was applied for non-normally distributed data. Associations between continuous variables were assessed using Spearman correlation. Comparisons between groups defined by dichotomous variables were analyzed using the Mann–Whitney U test. Results were reported as mean and standard deviation or as number and percentage. Statistical significance was set at *p* < 0.05 with a 95% confidence interval.

## 3. Results

### 3.1. Baseline Characteristics

**Figure 3:**
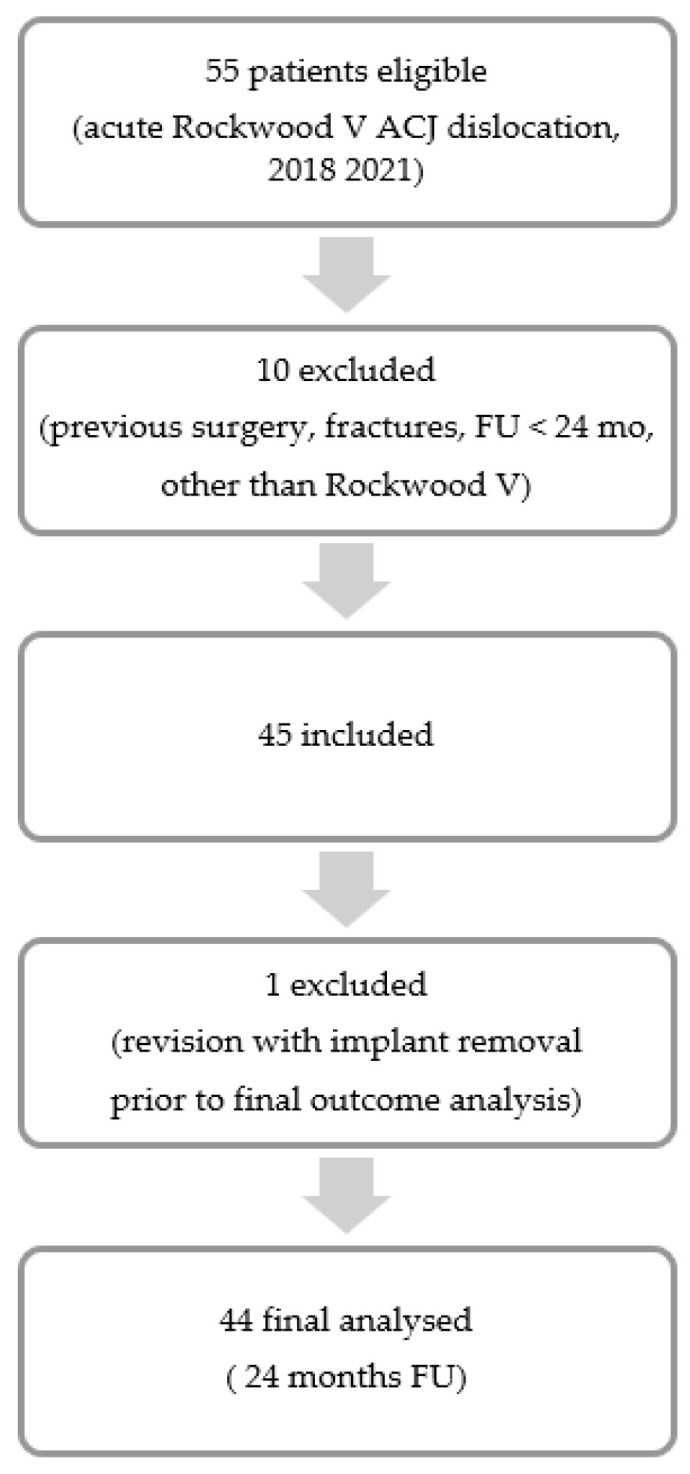
Patient selection flow.

Of 55 consecutive patients, a total of 45 patients met the inclusion criteria. One patient underwent revision surgery with implant removal six weeks postoperatively and was therefore excluded from the final analysis (Figure 3). The mean age of the final study population was 36.9 ± 9.8 years, and the mean follow-up period was 35.3 ± 8.5 months. A direct trauma mechanism was reported in 90.9% of cases, while 25.0% of patients presented with concomitant glenohumeral pathologies. The average interval between trauma and surgical intervention was 8.9 ± 4.3 days. Detailed patient characteristics are summarized in Table 1.

### 3.2. Clinical Results

At final follow-up, the CS averaged 89.6 ± 8.9 (range 51–100), the SSV was 91.1 ± 13.1 (range 30–100), and the TF was 9.9 ± 2.1 (range 4–12). The ACJI was 79.6 ± 15.5 (range 35–100). The VAS for pain upon palpation showed a mean of 0.9 ± 1.8 (range 0–10).

### 3.3. Radiological Results

The mean preoperative CC difference was 11.7 ± 2.8 mm, and the mean percentage difference compared to the contralateral side was 148.8% ± 46.8%. The mean postoperative CC difference was −2.6 ± 2.4 mm, which was on average measured 12.9 ± 17.8 days after surgery. At final follow-up, the mean CC difference was 2.0 ± 2.5 mm, with a mean percentage difference of 25.5 ± 31.6%. A total of 18.2% remained overreduced. According to the Rockwood classification, 47.7% were classified as type I, 27.3% as type II, and 27.3% as type III. In 2.3%, the difference exceeded 100%, corresponding to Rockwood type V (Table 2). Significant differences were observed in CC distance across preoperative, postoperative, and final follow-up measurements (χ^2^(2) = 84.182, *p* < 0.001), with all pairwise comparisons remaining significant (all *p* < 0.001). There was also a significant difference between the preoperative and final follow-up percentage differences (*p* < 0.001). The sub-button area significantly increased from 24.4 ± 7.5 mm^2^ at the postoperative measurements to 33.7 ± 11.2 mm^2^ at the final follow-up (t(43) = −6.628, *p* < 0.001, d = −0.999), while the mean tunnel diameter measured 6.0 ± 1.4 mm at the superior cortex, 5.5 ± 0.9 mm at the midsection, and 7.1 ± 1.8 mm at the inferior cortex. Ossifications between the clavicle and coracoid were observed in 84.1%. Osteoarthritis of the AC joint was present in 34.1%. DPT showed a partial dislocation in 31.8% and a complete dislocation in 2.3%. Detailed radiological results are summarized in Table 3.

### 3.4. Correlation Analysis

A positive correlation was found between the CC difference at the final follow-up and DPT (ρ = 0.341, *p* = 0.024). The CC difference at the final follow-up showed significant negative correlations with the TAFT score (ρ = −0.330, *p* = 0.028) and the ACJI (ρ = −0.320, *p* = 0.034). The percentage deviation at the final follow-up was positively correlated with horizontal translation (ρ = 0.367, *p* = 0.014) and negatively correlated with the ACJI (ρ = −0.334, *p* = 0.027). DPT demonstrated a significant negative correlation with the ACJI (ρ = −0.550, *p* < 0.001). VAS scores correlated negatively with both the SSV score (ρ = −0.350, *p* = 0.020) and the ACJI (ρ = −0.443, *p* = 0.003). No significant correlations were found between the CS and the radiological parameters. The sub-button area at the final follow-up showed only a significant negative correlation with the SSV score (ρ = −0.300, *p* = 0.048). There was no significant correlation between cTW and CC difference (ρ = –0.016, *p* = 0.918), percentage deviation (ρ = –0.019, *p* = 0.900), or DPT (ρ = 0.049, *p* = 0.750). Ossifications only showed significant positive correlations with the VAS score (ρ = 0.422, *p* = 0.004). Osteoarthritis correlated negatively with the ACJI (ρ = −0.375, *p* = 0.012). Moreover, the analyses revealed no significant differences in CS (*p* = 0.169), SSV (*p* = 0.936), TAFT (*p* = 0.936), ACJI (*p* = 0.237) scores, or VAS (*p* = 0.689) between patients with concomitant glenohumeral injuries and those without. Detailed radiological results are summarized in Table 4.

### 3.5. Complications

There was no implant-associated revision event. One patient underwent revision surgery due to a wound infection, which led to implant removal six weeks postoperatively.

## 4. Discussion

The principal finding of this study is that the described technique using the LPSB procedure, with additional AC cerclage fixation, yields high patient satisfaction without any revision events related to the hardware. To our knowledge, the present study comprises the largest case series treated with the LPSB implant. In a previous study, we found higher subjective satisfaction and no implant-associated revisions for the LPSB procedure compared to the double TightRope technique [31]. Based on these findings, the present study seeks to validate and further evaluate the value of this special implant in acute high-grade dislocations.

In the current literature, there is little evidence regarding the correct treatment strategy, timing of surgery and surgical procedure of choice. Low-grade AC joint dislocations are commonly treated conservatively, while high-grade dislocations are widely treated surgically [5,6,7]. When surgical treatment is performed, minimally invasive procedures have many advantages over open procedures such as concomitant diagnosis and treatment of glenohumeral injuries, minimization of soft tissue trauma, reduced risk of infection, better cosmetic results, higher subjective patient satisfaction and no second surgical intervention to remove the hardware [22,32,33]. Nevertheless, procedure-related complications have also been reported following arthroscopic fixation, such as hardware irritation, occurring in up to 35% of patients [22]. Glanzmann et al. reported on patients after double flip button stabilization, with 23.8% experiencing mild tenderness over the clavicular endobuttons and 4.8% severe irritation, considering surgical removal [34]. In a study by Scheibel et al., 39.3% of patients reported mild tenderness over the superior buttons and suture knots after stabilization with the first-generation TightRope implant [33]. Therefore, the LPSB implant is designed to minimize implant-associated discomfort by sinking the suture ends to avoid knot stacks above the superior button. A previous comparison between the singular LPSB technique and the DSB technique with two Dog Bone implants showed a significantly higher subjective satisfaction in favor of the LPSB patients. No patient in the LPSB group required revision surgery, while the DSB group showed a 7% revision rate due to implant irritation [31]. Also, in this study, no revision surgery was required for hardware irritation, with an overall SSV of 91.

The most common radiological complications are vertical and horizontal instability, drill-tunnel-related fractures and ossifications between the coracoid and the clavicle [22]. Biomechanical studies have shown that a minimally invasive double-button procedure, designed to restore the AC joint in an anatomic manner, achieves equal or even greater stability than the native coracoclavicular ligament complex [18,35]. Scheibel et al. reported on Rockwood type V dislocations treated with a double TightRope technique, yielding a CS of 92 and an SSV of 95%. However, 43% showed persistent horizontal instability on Alexander radiographs, which was associated with significantly lower TF and ACJI, while SSV and CS remained unaffected [33]. Further biomechanical research demonstrated that additional horizontal stabilization of the AC ligament complex improves overall stability [19,21,36]. Maziak et al. showed that patients who underwent isolated CC stabilization with a double bundle procedure were 4.8 times more likely to develop complete DPT than those who received additional AC stabilization [37]. At final follow-up, no significant correlation was found between DPT and SSV, but complete DPT was associated with increased residual pain in the ‘pain’ subcategory of both TF and ACJI [37]. In a previous comparison of the LPSB and the double TightRope technique, residual horizontal instability was observed in 28.6% and 32.1% of cases, respectively [31]. We observed instability in 34.1% of cases in total, with DPT showing only a significant negative correlation with the ACJI. Notably, the ACJI is the only clinical outcome measure that specifically assesses horizontal instability, while no significant effects of horizontal instability were observed on any of the other outcome scores. These substantial rates of instability are notable for both single- and double-bundle procedures, yet they seem to have no significant impact on clinical outcome. While this seems to represent merely a radiological finding, structural complications such as tunnel widening or clavicular fractures remain an important issue, with their risk increasing with the number of drill tunnels [23]. In a study by Bellmann et al., a high rate of cTW was observed following arthroscopically assisted AC joint stabilization using suture button devices, with up to 83% of patients affected depending on the implant type [38]. Tunnel area measurements revealed a significantly greater increase in tunnel diameter for the Dog Bone implant compared to the LPSB implant between 6 weeks and 6 months postoperatively. In patients treated with the Dog Bone implant, conical clavicular tunnel widening extended throughout the entire height of the clavicle, whereas in those treated with the LPSB implant, tunnel widening was strictly localized distal to the superior cortical button, where the suture material comes into direct contact with the bone [38]. In line with previous studies, this analysis demonstrated a high incidence of ossifications (84%) between the clavicle and the coracoid. However, this appeared to be of limited relevance, as only the VAS score showed a positive correlation, while all other clinical and radiological parameters remained unaffected, and no secondary interventions were required. Several studies have reported ossification rates of up to 68% without evidence of restrictions in shoulder motion [33,39]. Scheibel et al. reported heterotopic ossifications in 67.9% of cases and found that partial or complete bridging was associated even with significantly smaller CC distances, indicating greater vertical stability [33].

One major advantage of arthroscopic or arthroscopic-assisted surgical management over conservative treatment and open procedures is the ability to simultaneously diagnose and, if necessary, treat concomitant glenohumeral pathologies. Vetter et al. reported a prevalence of concomitant glenohumeral pathologies of 30.7% [40]. Supraspinatus tendon and labral lesions were the most commonly observed findings, and 69.2% of all cases were considered clinically relevant and required treatment. A higher prevalence of concomitant glenohumeral pathologies was associated with increasing patient age [40]. In a study by Jensen et al., concomitant glenohumeral pathologies were identified in 53% of patients with acute or chronic AC joint injuries of either Rockwood grade III or V [41]. Of those, 22% received an additional repair procedure [41]. Arrigoni et al. observed concomitant glenohumeral lesions in 42.8% of patients with ACJ dislocation, while 29.5% of these cases required additional surgical treatment [42]. GH injuries were present in 25.0% of patients in this study. We did not assess whether or how these injuries were treated, but their presence had no impact on clinical outcomes.

### Limitations

This study is limited by its retrospective design and the lack of a comparison group. Moreover, there was considerable variation in the timing of postoperative measurements. The 24-month follow-up period does not provide sufficient data to draw conclusions about long-term outcomes. Since there were no preoperative scores for the patients, calculation of the minimal clinically important differences was not possible. Moreover, a post hoc analysis (G*Power (Version 3.1.9.6), n = 44) showed a power of only 0.59 for medium effects, indicating that the nonsignificant regression results should be interpreted with caution. Nevertheless, the findings provide valuable exploratory insights and may guide future studies with larger cohorts.

## 5. Conclusions

Arthroscopic-assisted stabilization of acute high-grade AC joint dislocations using the LPSB technique with AC cerclage fixation provides excellent clinical outcomes and high patient satisfaction, with minimal implant-related complications and no need for revision surgery due to implant-related complications. Although cTW occurs, its clinical impact appears limited within this procedure.

## Figures and Tables

**Table 1:** Detailed baseline characteristics.

	Mean ± SD	Range
Age (years)	36.9 ± 9.8	19–61
Follow-up duration (months)	35.3 ± 8.5	24–55
Male Gender (%)	95.5%	
Direct Trauma mechanism (%)	90.9%	
Visible Concomitant GH ^a^ pathologies (%)	25.0%	
Dominant side involved (% yes)	32.0%	
Time from trauma to surgery (days)	8.9 ± 4.3	1–21

^a^ GH glenohumeral. Data are presented as mean, SD (±) and range or percent (%).

**Table 2:** Rockwood reclassification at the final follow-up.

Rockwood Type ^a^	Percentage
Rockwood Type I	47.7%
Rockwood Type II	27.3%
Rockwood Type III	27.3%
Rockwood Type V	2.3%
Overreduction	18.2%

^a^ Reclassification at the final follow-up based on the percentage deviation in comparison to the contralateral side. Data are presented as percent (%).

**Table 3:** Detailed radiological results.

	Mean ± SD	Range
Preoperative CC difference (mm)	11.7 ± 2.8	6.3–20.0
Preoperative percentage difference (%)	148.8 ± 46.9	100.0–309.7
Postoperative CC difference (mm)	−2.6 ± 2.4	−7.1–4.3
Final follow-up CC difference (mm)	2.0 ± 2.5	−3.6–7.1
Final follow-up percentage difference (%)	25.5 ± 31.6	−43.9–105.2
Sub-button area post-op (mm^2^)	24.4 ± 7.5	10.6–38.5
Sub-button area final follow-up (mm^2^)	33.7 ± 11.2	10.2–64.0
Tunnel diameter superior cortex (mm)	6.0 ± 1.4	4.9–12.0
Tunnel diameter midsection (mm)	5.5 ± 0.9	4.4–9.6
Tunnel diameter inferior cortex (mm)	7.1 ± 1.8	3.4–12.2
Ossifications present (%)	84.1%	
Osteoarthritis present (%)	34.1%	
DPT ^a^ stable (%)	65.9%	
DPT ^a^ partial dislocation (%)	31.8%	
DPT ^a^ complete dislocation (%)	2.3%	

^a^ DPT Dynamic Posterior Translation. Data are presented as mean, SD (±) and range or percent (%).

**Table 4:** Detailed results of the correlation analysis.

Clinical Scores	CC Difference	Percentage Difference	DPT	Sub-Button Area	cTW	Ossifications	Osteoarthritis
TAFT score	ρ = −0.330 *, *p* = 0.028	ρ = −0.266, *p* = 0.081	ρ = −0.269, *p* = 0.078	ρ = −0.117, *p* = 0.449	ρ = −0.004, *p* = 0.980	ρ = −0.004, *p* = 0.979	ρ = −0.256, *p* = 0.094
ACJI	ρ = −0.320 *, *p* = 0.034	ρ = −0.334 *, *p* = 0.027	ρ = −0.550 **, *p* < 0.001	ρ = −0.128, *p* = 0.408	ρ = −0.011, *p* = 0.944	ρ = −0.262, *p* = 0.086	ρ = −0.375 *, *p* = 0.012
CS	ρ = 0.112, *p* = 0.471	ρ = 0.148, *p* = 0.339	ρ = 0.132, *p* = 0.393	ρ = −0.126, *p* = 0.417	ρ = 0.199, *p* = 0.196	ρ = −0.062, *p* = 0.689	ρ = 0.076, *p* = 0.625
SSV score	ρ = 0.171, *p* = 0.266	ρ = 0.245, *p* = 0.110	ρ = −0.127, *p* = 0.413	ρ = −0.300 *, *p* = 0.048	ρ = 0.046, *p* = 0.765	ρ = −0.097, *p* = 0.532	ρ = −0.278, *p* = 0.068
VAS score	ρ = −0.051, *p* = 0.744	ρ = −0.060, *p* = 0.698	ρ = −0.240, *p* = 0.117	ρ = 0.055, *p* = 0.725	ρ = −0.029, *p* = 0.851	ρ = 0.422 **, *p* = 0.004	ρ = 0.416 **, *p* = 0.005

CC coracoclavicular distance at follow-up. DPT Dynamic Posterior Translation. ACJI Acromioclavicular Joint Instability Score. CS Constant Score. SSV Subjective Shoulder Value. VAS Visual Analogue Scale. TAFT Taft Score. cTW clavicular tunnel width. * *p* < 0.05, ** *p* < 0.01.

## Data Availability

The data presented in this study are available on reasonable request from the corresponding author. The data are not publicly available due to privacy restrictions.
